# The influence of self-relevance under time pressure on moral decision-making

**DOI:** 10.3389/fpsyg.2026.1778803

**Published:** 2026-05-21

**Authors:** Yang Zhang, Zijun Huang

**Affiliations:** 1 Cognition and Human Behavior Key Laboratory of Hunan Province, School of Education Science, Hunan Normal University, Changsha, China; 2 College of Marxism, Hunan Normal University, Changsha, China

**Keywords:** helping behavior, moral behavior, moral decision-making, self-relevance, time pressure

## Abstract

Moral decision-making is a core cognitive process that influences human social behavior. Understanding its underlying psychological mechanisms is crucial for comprehending individuals' prosocial tendencies and social adaptation. This study employs a situational priming paradigm to systematically investigate the impact of self-relevance on moral decision-making under time pressure. A pilot study assessed time pressure and established decision time thresholds for moderate and high levels of time pressure. Building on this, Experiment 1 examined the impact of time pressure (none, moderate, high) on moral decision-making, revealing that participants' helping behavior significantly increased under moderate and high time pressure compared to no time pressure. Experiment 2 introduced self-relevance to explore the joint effects of time pressure (none, moderate, high) and self-relevance (low, moderate, high) on moral decision-making. The results indicated that: (1) the main effect of self-relevance was significant, with helping behavior notably higher under high self-relevance than under low and moderate conditions; (2) within each self-relevance condition, helping behavior under moderate and high time pressure was significantly greater than under no time pressure; (3) under high self-relevance, participants maintained a similarly high level of helping behavior across all time pressure conditions (none, moderate, high). These findings suggest that time pressure facilitates altruistic moral decision-making in helping dilemmas. Moreover, individuals in high self-relevance condition tend to exhibit a greater proportion of helping behavior and are relatively less influenced by variations in time pressure. This study offers a novel perspective on how time pressure influences moral behavior within close relationships.

## 1 Introduction

Unethical behavior frequently occurs in real life, eroding social trust and undermining fairness. The intersection of psychology, cognitive neuroscience, and behavioral economics has made the factors influencing moral decision-making and their underlying mechanisms a central focus for researchers across multiple disciplines (Crockett et al., 2014; Zhan et al., 2022). In an increasingly fast-paced society, time has emerged as a significant situational variable that profoundly affects individuals' daily decision-making (Maruping et al., 2015; Wang et al., 2025). Exploring the directional effects and mechanisms of time pressure on moral decision-making holds both theoretical value and practical significance (Du et al., 2018; Suter and Hertwig, 2011). Importantly, existing research has confirmed that the social distance between decision-makers and the subjects of their decisions significantly moderates moral decision-making (Christensen and Gomila, 2012; Zhan et al., 2020). Self-relevance, as a core dimension of social distance, may influence individuals' moral judgment tendencies under time pressure (Singer et al., 2021; Yang et al., 2022). However, most existing studies default to using neutral strangers as decision subjects, often overlooking the critical factor of self-relevance. Thus, whether the impact of time pressure on moral decision-making varies with the degree of self-relevance remains to be empirically tested. This study focuses on moral decision-making dilemmas in helping scenarios, examining the impact of time pressure on moral decision-making and exploring the potential moderating role of self-relevance. We hope this research will support the theoretical framework for understanding moral decision-making and provide empirical evidence for the conditions under which moral behavior occurs in a fast-paced societal context.

### 1.1 Moral decision-making in dilemmas

Moral decision-making is a typical form of social decision-making that involves weighing self-interest against the interests of others. It refers to the psychological process by which individuals, faced with conflicting behavioral options in moral dilemmas, make optimal choices guided by social norms and their self-value orientation (Rilling and Sanfey, 2011). Social interactions are rife with such dilemmas, where individuals must make difficult choices between maximizing their own benefits and protecting the interests of others (Rand and Nowak, 2013). This process often accompanies intense negative emotional experiences and cognitive conflicts (Pletti et al., 2015; Zhan et al., 2022). The classic dual-process theory posits that individuals' moral cognition relies on both a rapid, unconscious, automatic “emotional intuition” system and a slower, conscious, controlled “deliberative cognition” system (Greene et al., 2001, 2004; Xu et al., 2020). In specific decision-making contexts, the extent to which emotional and cognitive factors are involved varies, with distinct neural networks in the brain for intuitive processing and cognitive reasoning dynamically balancing and competing with each other, ultimately leading to the formation of moral judgments (Goodwin and Darley, 2012; Paxton et al., 2012).

Previous studies often employed extreme hypothetical scenarios, such as the “trolley problem” or “bridge dilemma,” to examine individuals' moral judgments through forced-choice tasks. However, these traditional paradigms have faced criticism for being detached from real-life situations, as participants often perceive them as thought experiments lacking realistic engagement, limiting their ecological validity (Bauman et al., 2014; Gold et al., 2014). Researchers have thus called for increasing the realism and relevance of moral dilemmas, advocating for the examination of moral decision-making through both prescriptive morality (e.g., helping behavior) and proscriptive morality (e.g., harmful behavior) (Carnes and Janoff-Bulman, 2012; Noval and Stahl, 2017). In the realm of proscriptive morality, individuals must decide whether to actively deceive or harm others for personal gain (e.g., the “electric shock—profit” task). In contrast, prescriptive morality centers on whether individuals are willing to sacrifice their interests to help others (Crockett et al., 2014; Zhan et al., 2019). Typically, failing to assist those in distress is viewed as indifference, while actively harming others for profit is considered intolerably immoral, reflecting significant differences in the psychological mechanisms underlying these behaviors (Zhan et al., 2022). Given the positive implications of prescriptive morality for understanding individuals' prosocial tendencies and social adaptation, this study focuses on prescriptive moral forms, examining moral decision-making dilemmas in everyday helping situations and employing the more ecologically valid “moral dilemma situational priming paradigm” to systematically investigate the mechanisms of moral decision-making (Zhan et al., 2020; Zhong et al., 2017).

### 1.2 Time pressure and moral decision-making

In a complex and uncertain reality, individuals often need to make moral choices under urgent circumstances or strict time constraints. Time pressure refers to a psychological sense of urgency arising from time limitations or impending deadlines (Maruping et al., 2015; Wang et al., 2025). As an immediate situational cue, time pressure significantly influences individuals' moral judgments. In classic moral dilemmas, decision-making tendencies are primarily divided between deontology, which adheres to moral rules (i.e., harming others is unacceptable regardless of the outcome), and utilitarianism, which seeks to maximize overall benefits (i.e., harm is acceptable if it serves to maximize the welfare of the majority) (Du et al., 2018; Greene et al., 2001). The dual-process theory of moral decision-making suggests that when emotional processing dominates, individuals tend to make deontological decisions, while cognitive processing leads to utilitarian choices (Greene, 2007). Research indicates that under time pressure, individuals' cognitive resources are limited, placing them in a state of “cognitive busyness,” where increased processing load forces the decision-making process to rely more on fast, automated intuitive systems (Ferreira et al., 2006; Rosas and Aguilar-Pardo, 2020). Consequently, when faced with high time pressure, decision-makers often lack the opportunity for rational utilitarian calculations, exhibiting a greater tendency toward intuitive deontological judgments (Du et al., 2018; Fan et al., 2022; Suter and Hertwig, 2011). However, recent studies have yielded inconsistent conclusions, suggesting that the mechanisms of time pressure on moral decision-making may be more complex than initially expected (Del Popolo Cristaldi et al., 2024).

Notably, previous research has primarily focused on “sacrificial moral dilemmas” (e.g., deciding whether to sacrifice one person to save many in the trolley problem). These extreme hypothetical situations involving life and death are often viewed as thought experiments lacking real-world engagement, limiting their ecological validity (Bauman et al., 2014; Gold et al., 2014; Xu et al., 2020). Qmore, it remains unclear whether differences in dilemma types obscure the true effects of time pressure (Del Popolo Cristaldi et al., 2024). Given the limitations of extreme dilemmas, this study shifts its focus to more practically relevant everyday helping dilemmas. Helping behavior represents a typical form of prosocial decision-making. Existing research generally confirms that high time pressure and cognitive load significantly increase the incidence of cooperative behavior (Rand, 2016; Schulz et al., 2014; Yamagishi et al., 2017). Compared to time delays, time pressure not only directly increases individuals' helping behavior (Artavia-Mora et al., 2017) but also correlates positively with perceived urgency, where stronger time pressure and shorter decision times lead to higher levels of cooperation (Wang et al., 2022). Additionally, individuals' daily stress levels are positively correlated with their prosocial preferences, particularly in urgent situations characterized by compliance demands, anonymity, or high emotional arousal (Memmott-Elison et al., 2022; Yang et al., 2022). Based on this, we hypothesize that in everyday helping dilemmas, when time pressure is activated, individuals are more likely to make altruistic moral decisions. Therefore, we propose the following hypothesis:

***Hypothesis 1***: Compared to no time pressure, participants may exhibit a higher proportion of helping behavior under moderate and high time pressure.

### 1.3 Self-relevance and moral decision-making

In real life, individuals often face moral decisions involving not only strangers but also relatives, friends, and acquaintances in distress. This phenomenon has sparked discussions on the concept of self-relevance, which refers to the degree of association between moral situations or stimuli and the decision-maker (Christensen and Gomila, 2012; Zhang et al., 2024). Numerous studies have shown that self-relevance significantly moderates individuals' moral evaluations and behavioral tendencies: the closer the interpersonal distance, the more likely individuals are to exhibit prosocial intentions and altruistic behaviors (Chen et al., 2009; Cheng et al., 2010; Everett et al., 2015). Specifically, compared to strangers, individuals are more willing to sacrifice their interests to help relatives and friends (Miller and Bersoff, 1998), demonstrating greater empathy (Song et al., 2016), trust, and cooperation in social dilemmas (Chen et al., 2017). They are also more inclined to incur personal losses to reduce harm to close others (Zhan et al., 2022). This pattern holds in moral dilemmas involving helping, where individuals tend to make more altruistic decisions requiring personal sacrifice for friends compared to acquaintances and strangers (Zhan et al., 2019, 2020). From an evolutionary psychology perspective, this tendency aligns with the theory of kin selection, which posits that individuals may forgo their interests to assist relatives and friends, thereby maintaining genetic ties and ensuring the continuity of their genes (Hamilton, 1964; Tan et al., 2015).

Previous research has primarily examined the independent effects of time pressure and self-relevance on moral decision-making, with few studies exploring how these two factors interact. However, existing literature provides important clues regarding their joint mechanisms: empirical studies indicate that the influence of stress on altruistic moral decision-making induced by time pressure is significant only when decision subjects are close others (Singer et al., 2021; Yang et al., 2022), suggesting that time pressure may be moderated by self-relevance. Furthermore, individuals typically spend less time weighing pros and cons in moral decisions involving relatives or friends, experience smaller negative emotional conflicts, and expend fewer cognitive resources, leading to more altruistic choices (Zhan et al., 2018; 2020). Self-relevance also affects empathy levels: under high self-relevance condition, participants exhibit higher levels of empathy (Zhong et al., 2017), and their empathy for close others significantly exceeds that for strangers (Meyer et al., 2013; Song et al., 2016). This implies that when making moral decisions involving friends, the emotional/experiential system may be more pronounced, while decisions regarding strangers tend to rely more on cognitive processing. Integrating the dual-process theory of moral decision-making, when emotional processing dominates, individuals are more likely to make deontological decisions (Greene, 2007). High time pressure also limits cognitive resources, forcing decision-makers to rely on intuition for deontological judgments (Du et al., 2018; Fan et al., 2022; Suter and Hertwig, 2011). Thus, high self-relevance and high time pressure may synergistically promote altruistic moral decisions. These results suggest that self-relevance may moderate the influence of time pressure on moral decision-making, indicating that under high self-relevance condition, the effect of time pressure on altruistic moral decision-making may be more pronounced. Based on this, we propose the following hypotheses:

***Hypothesis 2a***: Compared to low and moderate self-relevance conditions, participants may exhibit a higher proportion of helping behavior under high self-relevance condition.

***Hypothesis 2b***: Self-relevance may moderate the effect of time pressure on moral decision-making, specifically under high self-relevance condition, where the effect of time pressure on increasing helping behavior may be stronger.

### 1.4 Overview of the study

In social interactions, time constraints are one of the significant determinants of human moral judgment and decision-making. Previous research has primarily examined the effects of time pressure or self-relevance on moral decision-making behavior in isolation, but it remains unclear how these two factors interactively influence moral decision-making, particularly in prescriptive morality (e.g., helping behaviors). Therefore, this study adopts the “moral dilemma situational priming paradigm” and designs three progressive experiments: the pilot study preliminarily assesses time pressure to determine decision time thresholds for moderate and high time pressure; Experiment 1 examines the impact of time pressure on moral decision-making to test Hypothesis 1; Experiment 2 investigates the joint effects of time pressure and self-relevance on moral decision-making to test Hypotheses 2a and 2b. This study aims to systematically reveal the key influencing factors of moral decision-making in helping dilemmas, providing empirical support for deepening classic theories in the field of morality and offering scientific evidence for moral behavior education.

## 2 Pilot study

### 2.1 Purpose

The pilot study aims to measure participants' average response time and standard deviation while reading moral dilemma scenarios. This measurement will help determine specific time settings that induce time pressure, thereby establishing decision times for moderate and high time pressure conditions in subsequent formal experiments.

### 2.2 Methods

#### 2.2.1 Participants

Forty participants (average age: 21.03 years; 24 females and 16 males) were recruited from a university in China. All participants were right-handed, had normal or corrected vision, and had not participated in similar experiments before. Informed consent was obtained from all participants, and they received small gifts upon completion of the experiment. A sensitivity power analysis indicated that this sample size was sufficient to detect an effect size of *f* = 0.25 with a statistical power of 0.80 at a significance level of α = 0.05 (G^*^Power 3.1; Faul et al., 2007).

#### 2.2.2 Experimental materials

The moral dilemma scenarios were adapted from previous research and designed based on the methodology developed by Loke et al. (2011), consisting of 30 common and relatable helping dilemmas (see Appendix). Each dilemma includes a scenario description and two options: in the scenario, the protagonist is in danger and needs help, while the participant is engaged in an important task and must make an immediate decision about whether to provide help. One option describes an “altruistic moral decision,” which involves abandoning one's important task to offer help immediately; the other option describes a “selfish moral decision,” which entails continuing with one's own task and ignoring the request for help. The word count and familiarity of each scenario were carefully controlled, with each scenario containing fewer than 100 words and each option approximately 20–30 words. These materials have been confirmed as effective in previous research (Zhan et al., 2020).

#### 2.2.3 Experimental procedure

Participants entered a quiet laboratory sequentially to participate in the experiment. The experimental procedure was programmed and presented using E-Prime 2.0. Participants read 30 moral dilemma scenarios on a computer, with the presentation order of all scenarios fully randomized. After reading each scenario, participants were required to immediately press a key to proceed to the next question, which prevented deep contemplation, while a timer recorded the reading time for each scenario.

### 2.3 Results

Based on previous research on criteria for time pressure (Du et al., 2018), if individuals' decision-making times follow a normal distribution, setting a limit time at one standard deviation below the average decision time will result in 84% of participants lacking sufficient time to make decisions. Therefore, a value below one standard deviation from the average time serves as the standard for time pressure. In this experiment, the decision times of all participants generally conformed to a normal distribution. Previous experiments often measured time pressure using two levels: with and without. However, this experiment introduced a moderate time pressure level as a transitional variable to explore the impact of time pressure on moral decision-making more deeply. The specific assessment method was as follows: First, the average response time and standard deviation for the 40 participants reading the 30 moral dilemma scenarios were determined. The average response time minus one standard deviation was then used as the setting for moderate time pressure. Next, the moderate time pressure setting minus one standard deviation was used as the setting for high time pressure. Ultimately, the moderate time pressure setting was established at 3,000 milliseconds (ms), and the high time pressure setting was established at 1,900 ms.

## 3 Experiment 1

### 3.1 Purpose and hypothesis

This experiment aims to employ the moral dilemma situational priming paradigm to examine the effect of time pressure on moral decision-making, testing Hypothesis 1.

### 3.2 Methods

#### 3.2.1 Participants

One hundred fifty participants (average age: 23.61 years; 88 females and 62 males) were recruited from a university in China. All participants were right-handed, had normal or corrected vision, and had not participated in similar experiments before. Informed consent was obtained, and participants received small gifts upon completion of the experiment. The 150 participants were randomly assigned to three groups: no time pressure, moderate time pressure, and high time pressure, with 50 participants in each group. A sensitivity power analysis indicated that this sample size was sufficient to detect an effect size of *f* = 0.25 with a statistical power of 0.80 at a significance level of α = 0.05 (G^*^Power 3.1; Faul et al., 2007).

#### 3.2.2 Experimental design

A between-subjects experimental design with a single factor at three levels (time pressure: no time pressure vs. moderate time pressure vs. high time pressure) was employed. The dependent variable was the proportion of help chosen by participants in the moral dilemma situational priming paradigm (i.e., helping proportion).

#### 3.2.3 Experimental materials

1. **Moral dilemma scenarios**
  The same moral dilemma scenarios used in the pilot study were employed, consisting of 30 common and relatable helping dilemmas.
2. **Time pressure questionnaire**
  Based on the results of the pilot study, decision times under three levels of time pressure were set as follows: no time pressure allowed for unlimited decision time; under moderate time pressure, the decision time was limited to 3,000 ms; and under high time pressure, the decision time was limited to 1,900 ms. After initiating time pressure, a time pressure questionnaire was used to measure the perceived time pressure experienced post-initiation, validating the manipulation of time pressure. This questionnaire consisted of eight items (e.g., “I feel that time is passing quickly”), rated on a 5-point Likert scale (1 representing “strongly disagree” and 5 representing “strongly agree”), where higher scores indicated greater time pressure (Wang and Hu, 2008).

#### 3.2.4 Experimental procedure

Before the formal experiment, participants were randomly assigned to the no time pressure, moderate time pressure, or high time pressure groups to ensure balanced group sizes. Participants then entered a quiet laboratory to participate in the experiment. The experimental procedure was programmed and presented using E-Prime 2.0. At the start of the experiment, participants were informed that they would see different scenario questions, after which they entered the decision interface and made selections using the keyboard: the “*F*” key indicated helping others, and the “*J*” key indicated not helping. Five practice scenarios were provided to help participants understand the experimental process.

**Figure 1:**
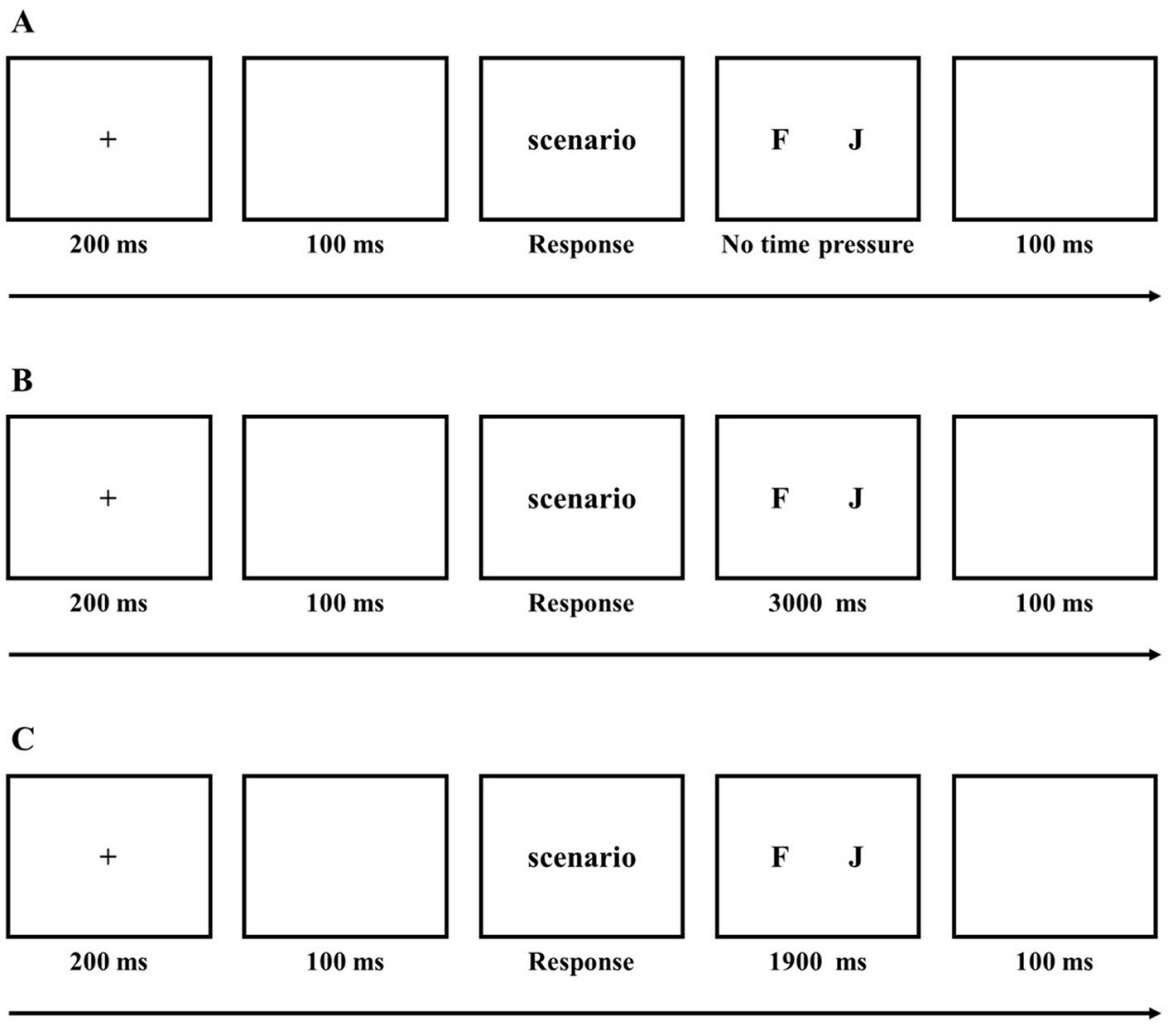
Flowchart of Experiment 1

For the no time pressure group, a fixation point was presented for 200 ms, followed by a blank screen for 100 ms. Next, the scenario was presented, and participants could press the space bar to proceed to the next screen after reading. The decision interface was then presented, where participants made their decisions by pressing the corresponding keys: “*F*” for helping and “*J*” for not helping, with no time limit on this interface. After key selection, a blank screen was shown for 100 ms. There were a total of 30 scenarios, each constituting one trial, resulting in 30 trials (see Figure 1A).

For the moderate time pressure group, the procedure was the same as for the no time pressure group, with the only difference being that the decision interface had a time limit of 3,000 ms (see Figure 1B).

For the high time pressure group, the procedure was the same as for the moderate time pressure group, with the only difference being that the decision interface had a time limit of 1,900 ms (see Figure 1C).

### 3.3 Results

#### 3.3.1 Manipulation check for time pressure

A one-way ANOVA was conducted on the average scores of the time pressure questionnaire across the three groups. The results indicated significant differences in the mean scores of the time pressure questionnaire among the three groups, *F*(2, 147) = 15.21, *p* < 0.001, $\eta_{p\;}^{2}\;$ = 0.060. Post-hoc multiple comparisons revealed that the high time pressure group's mean score was significantly higher than that of the moderate time pressure group, *p* < 0.05, and also significantly higher than that of the no time pressure group, *p* < 0.001; additionally, the mean score of the moderate time pressure group was also significantly higher than that of the no time pressure group, *p* < 0.001. These results confirm that the manipulation of time pressure was effective.

#### 3.3.2 Helping proportion

**Figure 2:**
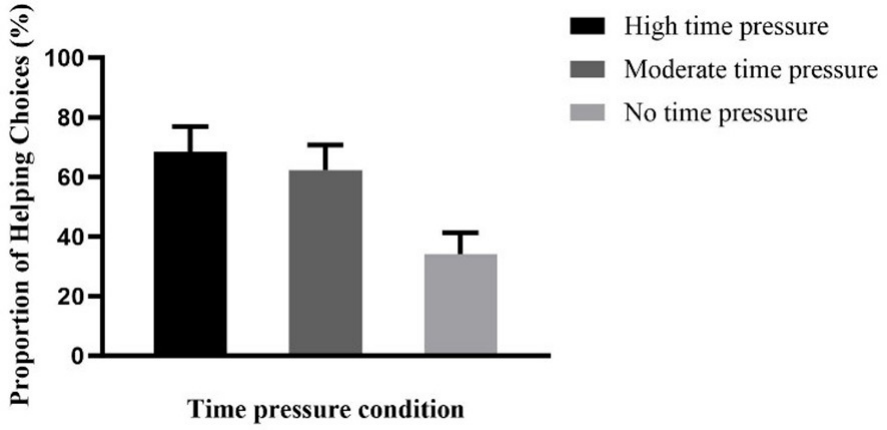
Helping proportion for high, moderate, and no time pressure groups in Experiment 1

A one-way ANOVA was conducted on the helping proportion of participants in the no, moderate, and high time pressure groups. The results showed a significant main effect of time pressure, *F*(2, 147) = 170.97, *p* < 0.001, $\eta_{p\;}^{2}=$ 0.342. Post-hoc multiple comparisons indicated that the helping proportion in the high time pressure group was significantly higher than that in the moderate time pressure group, *p* < 0.05, and also significantly higher than that in the no time pressure group, *p* < 0.001; additionally, the helping proportion in the moderate time pressure group was also significantly higher than that in the no time pressure group, *p* < 0.001 (see Table 1 and Figure 2).

**Table 1:** Mean and standard deviation of helping proportion for no, moderate, and high time pressure groups in Experiment 1.

High time pressure	Moderate time pressure	No time pressure
*M ± SD*	*M ± SD*	*M ± SD*
68.52% ± 8.52%	62.34% ± 8.40%	34.21% ± 7.12%

### 3.4 Discussion of experiment 1

The results of Experiment 1 indicated that participants exhibited a significantly higher proportion of helping behavior under both moderate and high time pressure compared to the no time pressure condition, with the helping proportion under high time pressure significantly exceeding that under moderate time pressure. This finding supports Hypothesis 1 and is consistent with previous research. Studies have shown that, under time pressure, individuals tend to rely more on fast, automatic intuitive systems due to a state of “cognitive busyness” (Ferreira et al., 2006; Rosas and Aguilar-Pardo, 2020). This reliance may lead them to forgo rational utilitarian calculations in favor of intuitive moral judgments (Du et al., 2018; Suter and Hertwig, 2011). Furthermore, time pressure not only increases helping behaviors (Artavia-Mora et al., 2017) but is also positively correlated with prosocial preferences (Memmott-Elison et al., 2022). Thus, under moderate or high time pressure, individuals may spend less time weighing personal gains and losses, opting instead to help others in moral dilemmas.

While Experiment 1 demonstrated that time pressure promotes altruistic moral decision-making, it primarily involved strangers as protagonists. In real-life scenarios, friends and acquaintances often face difficult circumstances. Previous research has indicated that self-relevance influences moral behavior: the higher the self-relevance, the more likely individuals are to exhibit prosocial intentions and altruistic behavior (Cheng et al., 2010; Zhan et al., 2020). However, past studies have largely examined the independent effects of time pressure and self-relevance on moral decision-making, with limited exploration of their interaction. Therefore, Experiment 2 will further investigate the joint impact of time pressure and self-relevance on moral decision-making, aiming to reveal whether the effect of time pressure on moral decisions varies according to differences in self-relevance.

## 4 Experiment 2

### 4.1 Purpose and hypothesis

This experiment aims to employ the moral dilemma situational priming paradigm to examine the effects of time pressure and self-relevance on moral decision-making, testing Hypotheses 2a and 2b.

### 4.2 Methods

#### 4.2.1 Participants

One hundred twenty participants (average age: 21.26 years; 52 females and 68 males) were recruited from a university in China. All participants were right-handed, had normal or corrected vision, and had not participated in similar experiments before. Informed consent was obtained, and participants received small gifts upon completion of the experiment. The 120 participants were randomly assigned to three groups: no time pressure, moderate time pressure, and high time pressure, with 40 participants in each group. A sensitivity power analysis indicated that this sample size was sufficient to detect an effect size of *f* = 0.25 with a statistical power of 0.80 at a significance level of α = 0.05 (G^*^Power 3.1; Faul et al., 2007).

#### 4.2.2 Experimental design

A mixed experimental design with 3 (time pressure: no time pressure vs. moderate time pressure vs. high time pressure) × 3 (self-relevance: low self-relevance vs. moderate self-relevance vs. high self-relevance) was employed. Time pressure served as the between-subjects factor, while self-relevance served as the within-subjects factor. The dependent variable was the same as in Experiment 1.

#### 4.2.3 Experimental materials

1. **Moral dilemma scenarios**
  Similar to Experiment 1, the same moral dilemma scenarios used in the pilot study were employed, consisting of 30 common and relatable helping dilemmas.
2. **Time pressure questionnaire**
  The time pressure questionnaire was used to measure perceived time pressure post-induction, similar to Experiment 1, to validate the manipulation of time pressure.
3. **Self-relevance stimuli and IOS scale**
  Following previous research, this experiment employed three categories of target others' names (i.e., friends, acquaintances, strangers) as self-relevance induction stimuli (Zhan et al., 2020). Before the experiment, participants were asked to provide the names of one same-gender friend and one acquaintance with the same number of characters and to rate the familiarity of these name stimuli. A friend was defined as “a same-gender friend with whom one has interacted frequently and consistently for over 3 years,” while an acquaintance was defined as “a same-gender classmate or peer with whom one has a nodding acquaintance for over 3 years.” A stranger was represented by an experimenter from the laboratory. Additionally, the Inclusion of Others in the Self (IOS) scale was used to assess the self-relevance level of target others, with scores of 5–7 indicating high self-relevance (e.g., friends), scores of 3–4 indicating moderate self-relevance (e.g., acquaintances), and scores of 1–2 indicating low self-relevance (e.g., strangers) (Aron et al., 1992; Zhan et al., 2020). Self-relevance was manipulated by substituting the names of target others for the names of protagonists in the moral dilemma scenarios (e.g., friend—Zhang San, acquaintance—Li Si, or stranger—Wang Wu).

#### 4.2.4 Experimental procedure

Before the formal experiment, participants were randomly assigned to the no time pressure, moderate time pressure, or high time pressure groups to ensure balanced group sizes. Participants then entered a quiet laboratory to participate in the experiment. The experimental procedure was programmed and presented using E-Prime 2.0. At the start of the experiment, participants were informed that they would see different scenario questions, after which they entered the decision interface and made selections using the keyboard: the “F” key indicated helping others, and the “J” key indicated not helping. Five practice scenarios were provided to help participants understand the experimental process.

**Figure 3:**
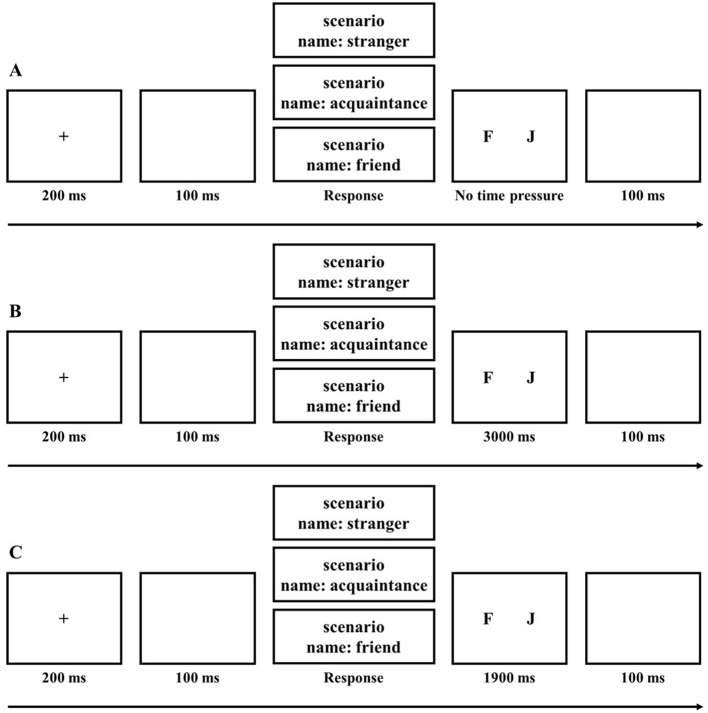
Flowchart of Experiment 2

For the no time pressure group, a fixation point was presented for 200 ms, followed by a blank screen for 100 ms. Next, the scenario was presented with self-relevant names (corresponding to friends for high self-relevance, acquaintances for moderate self-relevance, and strangers for low self-relevance). The scenarios with the three types of names were presented randomly, and participants could press the space bar to proceed to the next screen after reading the scenario. Subsequently, the decision interface was presented, where participants made their decisions by pressing the corresponding keys: “*F*” for helping and “*J*” for not helping, with no time limit on this interface. After key selection, a blank screen was shown for 100 ms. There were three self-relevance conditions, each with 30 scenarios, resulting in a total of 90 trials (see Figure 3A).

For the moderate time pressure group, the procedure was the same as for the no time pressure group, with the only difference being that the decision interface had a time limit of 3,000 ms (see Figure 3B).

For the high time pressure group, the procedure was the same as for the moderate time pressure group, with the only difference being that the decision interface had a time limit of 1,900 ms (see Figure 3C).

### 4.3 Results

#### 4.3.1 Manipulation check for time pressure

A one-way ANOVA was conducted on the average scores of the time pressure questionnaire across the three groups. The results indicated significant differences in the mean scores of the time pressure questionnaire among the three groups, *F*(2, 117) = 310.74, *p* < 0.001, $\eta_{p\;}^{2}$ = 0.906. Post-hoc multiple comparisons revealed that the mean score of the high time pressure group was significantly higher than that of the moderate time pressure group, *p* < 0.001, and also significantly higher than that of the no time pressure group, *p* < 0.001; additionally, the mean score of the moderate time pressure group was also significantly higher than that of the no time pressure group, *p* < 0.001. These results confirm that the manipulation of time pressure was effective.

#### 4.3.2 Manipulation check for self-relevance

A repeated measures ANOVA was conducted on the IOS scores for the three types of target others' names. The results indicated a significant main effect of self-relevance, *F*(2, 234) = 48.96, *p* < 0.001, $\eta_{p\;}^{2}$ = 0.210. Post-hoc multiple comparisons revealed that the IOS scores for friends' names were significantly higher than those for acquaintances' names, *p* < 0.001, and also significantly higher than those for strangers' names, *p* < 0.001; additionally, the IOS scores for acquaintances' names were also significantly higher than those for strangers' names, *p* < 0.001. These results confirm that the manipulation of self-relevance was effective.

#### 4.3.3 Helping proportion

A repeated measures ANOVA was conducted on the helping proportion with 3 (time pressure: no time pressure vs. moderate time pressure vs. high time pressure) × 3 (self-relevance: low self-relevance vs. moderate self-relevance vs. high self-relevance). The results indicated a significant main effect of time pressure, *F*(2, 117) = 94.54, *p* < 0.001, $\eta_{p\;}^{2}$ = 0.618. Post-hoc multiple comparisons revealed that the helping proportion in the high time pressure group was significantly higher than that in the moderate time pressure group, *p* < 0.05, and also significantly higher than that in the no time pressure group, *p* < 0.001. Additionally, the helping proportion in the moderate time pressure group was significantly higher than that in the no time pressure group, *p* < 0.01. The main effect of self-relevance was also significant, *F*(2, 234) = 3,222.21, *p* < 0.001, $\eta_{p\;}^{2}$ = 0.965. Post-hoc multiple comparisons showed that the helping proportion under high self-relevance condition was significantly higher than that under moderate self-relevance condition, *p* < 0.001, and also significantly higher than that under low self-relevance condition, *p* < 0.001. Furthermore, the helping proportion under moderate self-relevance condition was significantly higher than that under low self-relevance condition, *p* < 0.001.

**Figure 4:**
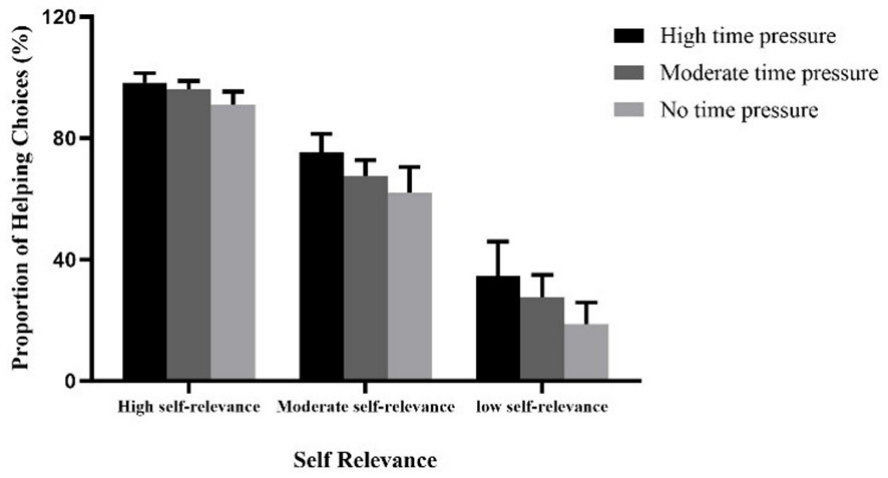
Helping proportion for high, moderate, and no time pressure groups under high, moderate, and low self-relevance conditions in Experiment 2

Moreover, the interaction between time pressure and self-relevance was significant, *F*(4, 234) = 5.19, *p* < 0.001, $\eta_{p\;}^{2}$ = 0.081. Simple effects analysis revealed that under high self-relevance condition, there were significant differences in the helping proportion across different levels of time pressure, *p* < 0.001. Post-hoc multiple comparisons indicated that the helping proportion in the high time pressure group was significantly higher than that in the moderate time pressure group, *p* < 0.05, and also significantly higher than that in the no time pressure group, *p* < 0.01. Additionally, the helping proportion in the moderate time pressure group was significantly higher than that in the no time pressure group, *p* < 0.05. Under moderate self-relevance condition, significant differences in helping proportion across different time pressures were also observed, *p* < 0.001. Post-hoc multiple comparisons showed that the helping proportion in the high time pressure group was significantly higher than that in the moderate time pressure group, *p* < 0.01, and also significantly higher than that in the no time pressure group, *p* < 0.001. Furthermore, the helping proportion in the moderate time pressure group was significantly higher than that in the no time pressure group, *p* < 0.01. Under low self-relevance condition, significant differences in helping proportion across different time pressures were similarly observed, *p* < 0.001. Post-hoc multiple comparisons revealed that the helping proportion in the high time pressure group was significantly higher than that in the moderate time pressure group, *p* < 0.01, and also significantly higher than that in the no time pressure group, *p* < 0.001. Additionally, the helping proportion in the moderate time pressure group was significantly higher than that in the no time pressure group, *p* < 0.001 (see Table 2 and Figure 4).

**Table 2:** Mean and standard deviation of helping proportion for three time pressure groups under different self-relevance conditions in Experiment 2.

Helping proportion	High time pressure	Moderate time pressure	No time pressure
	***M** **±** **SD***	***M** **±** **SD***	***M** **±** **SD***
High self-relevance	98.20% ± 3.22%	96.07% ± 2.79%	91.15% ± 4.18%
Moderate self-relevance	75.32% ± 6.10%	67.60% ± 5.19%	62.13% ± 8.34%
Low self-relevance	34.65% ± 11.26%	27.60% ± 7.34%	18.80% ± 7.09%

### 4.4 Discussion of experiment 2

The results of Experiment 2 indicated that participants exhibited a significantly higher helping proportion under moderate and high time pressure compared to the no time pressure condition, with the helping proportion under high time pressure also significantly exceeding that under moderate time pressure. This finding supports Hypothesis 1 and is consistent with the results of Experiment 1. Furthermore, participants demonstrated a significantly higher helping proportion in the high self-relevance condition compared to both low and moderate self-relevance conditions, while the helping proportion in the moderate self-relevance condition was also significantly higher than in the low self-relevance condition. This finding validates Hypothesis 2a and aligns with previous research showing that individuals are more willing to sacrifice their own interests to help family and friends (Miller and Bersoff, 1998). In moral dilemmas, individuals are more likely to make altruistic helping decisions for friends than for acquaintances or strangers (Zhan et al., 2019, 2020). Consequently, individuals are more inclined to extend a helping hand when friends are in distress.

Although Experiment 2 found an interaction effect between time pressure and self-relevance, the helping proportion under moderate and high time pressure remained significantly higher than under no time pressure across all self-relevance conditions. Furthermore, under high self-relevance condition, participants maintained a relatively high level of helping behavior under no, moderate, or high time pressure. This result diverges from Hypothesis 2b, which may be attributed to a strong preference for self-relevance in moral decision-making (Zhan et al., 2022; Zhang et al., 2024), aligning with the evolutionary significance of kin selection theory (Hamilton, 1964). Previous studies have shown that individuals tend to favor those with high self-relevance, such as close relatives, in moral decision-making, whether adopting a consequence-oriented utilitarian approach or a rule-oriented deontological approach (Feng et al., 2014). Consequently, individuals with high self-relevance generally receive assistance at a higher proportion. Under high self-relevance condition, individuals exhibit elevated levels of altruistic moral behavior and may be relatively less affected by fluctuations in time pressure. However, these speculations require further validation and exploration in future research.

## 5 General discussion

### 5.1 The impact of time pressure on moral decision-making

The results of Experiments 1 and 2 consistently indicated that participants exhibited a significantly higher proportion of helping behavior under moderate and high time pressure compared to no time pressure. This suggests that time pressure facilitates altruistic moral decision-making in helping dilemmas. According to dual-process theory, when emotional processing dominates, individuals tend to make deontological decisions, while cognitive processing leads to a preference for utilitarian choices (Greene, 2007). Under time pressure, cognitive resources become severely limited, resulting in cognitive overload. Consequently, individuals rely more on fast, automatic intuitive systems during decision-making (Ferreira et al., 2006; Rosas and Aguilar-Pardo, 2020). Thus, when faced with high time pressure, decision-makers often struggle to engage in rational, utilitarian weighing of self and others' interests, instead favoring intuitive moral judgments that prioritize not harming others (Du et al., 2018; Fan et al., 2022; Suter and Hertwig, 2011). Moreover, research on prosocial behavior has confirmed that time pressure enhances cooperative behavior (Wang et al., 2022) and directly increases helping behaviors (Artavia-Mora et al., 2017). Additionally, an individual's daily stress level is positively correlated with prosocial preferences, particularly in emergency situations or contexts characterized by high emotional arousal, where this positive effect is particularly pronounced (Memmott-Elison et al., 2022; Yang et al., 2022).

This study diverges from traditional moral decision-making research, which often focuses on extreme “sacrificial moral dilemmas” (Bauman et al., 2014; Gold et al., 2014; Xu et al., 2020), by primarily examining more realistically relevant everyday helping dilemmas. The findings indicate that when time pressure is effectively induced, individuals are more likely to make altruistic moral decisions in these everyday contexts. This enriches the application of dual-process theory in moral decision-making and provides new insights into individual moral behavior in emergency situations. As time pressure increasingly escalates in rapidly changing social environments, understanding its influence on moral decision-making is crucial for designing effective moral education programs that promote prosocial behavior. Notably, while this study highlights the facilitative effects of time pressure, it does not thoroughly explore whether a threshold exists at which the impact on altruistic moral decision-making saturates or stagnates, a question that warrants further investigation in future research.

### 5.2 The effects of self-relevance and time pressure on moral decision-making

The results of Experiment 2 revealed that participants were significantly more likely to help individuals with high self-relevance (friends) compared to those with moderate and low self-relevance (acquaintances and strangers). This indicates that higher self-relevance correlates with greater altruistic moral decision-making in helping dilemmas. From an evolutionary psychology perspective, this tendency to differentiate between close and distant relationships aligns with kin selection theory, which posits that individuals may sacrifice some self-interest to assist relatives and friends, thereby maintaining genetic ties and ensuring the continuity of their genes (Hamilton, 1964; Tan et al., 2015). Empirical research consistently shows that individuals are more likely to make altruistic decisions requiring self-sacrifice for friends compared to acquaintances and strangers (Zhan et al., 2019, 2020). People are generally more willing to sacrifice their own interests to help family and friends (Miller and Bersoff, 1998), demonstrating greater trust and cooperation in social dilemmas (Chen et al., 2017) and often incurring personal losses to reduce harm to close others (Zhan et al., 2022). This study not only enriches the application of kin selection theory in everyday moral decision-making but also provides insights into how individuals navigate moral choices within complex social relationships.

However, although Experiment 2 found an interaction effect between time pressure and self-relevance on helping behavior, the helping proportion under moderate and high time pressure remained significantly higher than that under no time pressure across all self-relevance conditions. Furthermore, under high self-relevance condition, participants maintained a relatively high level of helping behavior under no, moderate, or high time pressure. This suggests that individuals with high self-relevance generally receive help at a higher rate and may be relatively less affected by variations in time pressure. Previous research indicates that, in moral decision-making, individuals tend to favor those with high self-relevance, such as family members, regardless of whether they adopt a results-oriented utilitarian approach or a rule-based deontological approach (Feng et al., 2014). Therefore, individuals may engage in more helping behavior under high self-relevance condition, regardless of how time pressure influences their tendencies toward deontological or utilitarian decisions. Additionally, studies have found that when negative events occur to an individual's friends, these events can increase the individual's anxiety (Gao et al., 2022). Moral dilemmas that are more closely related to the individual typically provoke greater decision conflict and negative emotional experiences (Christensen and Gomila, 2012; Zhong et al., 2017). This may lead to increased anxiety due to a friend's suffering even in the absence of time pressure, prompting individuals to help friends across all levels of time pressure. Furthermore, this may also be attributed to a strong preference for self-relevance in human moral decision-making behavior (Zhan et al., 2022; Zhang et al., 2024), suggesting that altruistic moral decisions are likely to remain high under condition of high self-relevance, with the influence of time pressure variations being relatively weaker. Nevertheless, these speculations require further validation in future studies. This research provides a new perspective on how time pressure affects moral behavior in close relationships and offers important insights for moral education and intervention practices. Specifically, enhancing emotional connections between individuals may increase the incidence of altruistic behavior in moral education programs.

### 5.3 Limitations and future directions

This study systematically examined the impact of time pressure on moral decision-making in helping dilemmas while exploring the important role of self-relevance. Although the findings enrich relevant theories and provide significant references for future exploration, several limitations warrant attention in subsequent research:

First, this study employed a uniform standard to set decision times under moderate and high time pressure. While the text length and familiarity of each moral dilemma scenario were carefully controlled, individual differences in reading times may still exist across different scenarios. Future research could evaluate different scenarios individually and set time pressure levels that adapt to each scenario, thereby enhancing the precision of experimental manipulation.

Second, the moral dilemma scenarios used in this study were hypothetical and lacked real incentives or costs, which may limit the external validity of the results. Future research could introduce real monetary or time costs to ensure that participants' moral choices have substantial personal implications, effectively addressing the ecological validity shortcomings of hypothetical scenarios and further testing the external generalizability of the conclusions drawn from this study.

Lastly, this study primarily focused on prescriptive moral decision-making (e.g., helping behavior) while lacking exploration of proscriptive moral decision-making (e.g., harmful behavior). Additionally, the study mainly employed behavioral experimental methods and could not reveal the underlying neural mechanisms of moral decision-making. Thus, future studies could integrate neuroscience techniques to explore the decision-making differences between prescriptive and proscriptive morality, as well as their neural bases, to achieve a more comprehensive understanding of moral decision-making. Furthermore, future research could investigate the boundary conditions and mechanisms underlying the interaction between time pressure and self-relevance. This could be achieved by introducing more finely differentiated self-relevance manipulations or by examining additional potential moderating variables.

## 6 Conclusion

1. Time pressure has a facilitating effect on altruistic moral decision-making in helping dilemmas.
2. People generally exhibit a higher level of helping behavior toward individuals with high self-relevance, and this behavior may be relatively less influenced by variations in time pressure.

## Data Availability

The raw data supporting the conclusions of this article will be made available by the authors, without undue reservation.
