# Supplementary material for: The influence of self-relevance under time pressure on moral decision-making
Source: Front Psychol. 2026 May 21;17:1778803. doi: 10.3389/fpsyg.2026.1778803 (PMC13233455; doi:10.3389/fpsyg.2026.1778803)
Supplement: Supplementary file 1 [file Data_Sheet_1.pdf]

Appendix

Moral dilemma scenarios:

1. A person has been struck by a car while you are en route to take the graduate entrance exam. He urgently requires your assistance to reach the hospital.

F: If you help, you will forfeit years of exam preparation.

J: If you do not help, his life may be at risk.

2. During an audit, you discover that a colleague has embezzled funds. He pleads with you not to report him, citing his father's urgent need for money for surgery and promising to rectify the situation before the next audit.

F: If you assist him, you may face implications, including dismissal and criminal charges.

J: If you do not assist, he risks termination and legal consequences, and his father may not receive necessary treatment.

3. A robber has taken a hostage at the jewelry store where you work as a sales clerk. You have activated the alarm, but the robber threatens the hostage. Would you consider taking the hostage's place?

F: If you help, you may encounter life-threatening danger, as the robber may retaliate against you.

J: If you do not help, the hostage's life may be in jeopardy.

4. A person injured in a car accident requires immediate medical attention. You are driving to visit your wife, who is in labor, when a bystander implores you to transport the injured person to the nearest hospital.

F: If you help, you risk missing the birth of your child and being unavailable for your wife.

J: If you do not help, the injured individual may suffer life-threatening complications due to treatment delays.

5. While hiking alone on a remote mountain, you discover a climber who has sustained severe leg injuries. He requests your help to reach the nearest hospital.

F: If you assist him, you must abandon your expensive climbing gear, which he will not reimburse.

J: If you do not help, he may bleed to death.

6. A colleague diagnosed with acute leukemia urgently needs 20,000 yuan for surgery. You have just received half a year's salary and intended to send it home to support your family. He desperately

asks to borrow money for his treatment.

F: If you help, you may face financial difficulties in supporting your family.

J: If you do not help, his condition may deteriorate, endangering his life.

7. A survivor of a shipwreck calls for help from your boat, which is already full after escaping a disaster.

F: If you help, your boat may capsize.

J: If you do not help, he may lack the strength to survive until another boat arrives, putting his life in danger.

8. A financially disadvantaged student in your village has been accepted to university but urgently needs a substantial amount for tuition. As a village official who has just secured funds to repair a well, you find the villagers preoccupied with that project when he approaches you for assistance.

F: If you help, the well repairs may be delayed, and you may face backlash from the villagers.

J: If you do not help, he may miss the opportunity for education, jeopardizing his future.

9. A person severely injured by a motorcycle at night seeks your assistance. As a fruit vendor relying on daily sales to support your family, he asks you to take him to the nearest hospital for immediate treatment.

F: If you help, you will incur a loss of income, and he will not compensate you.

J: If you do not help, he may suffer permanent disability due to delayed treatment.

10. After a gas explosion at home, a person is seriously injured and rushed into your taxi by his family. To save time, he asks you to run a red light.

F: If you help, you risk losing your driver's license due to traffic violations, which would prevent you from driving a taxi again.

J: If you do not help, he may die due to delays in receiving treatment.

11. A person collapses from a cerebral hemorrhage on the roadside. As a renowned surgeon on your way to deliver an important international lecture, he requests immediate assistance to reach the hospital.

F: If you help, you will miss the lecture, potentially impacting your career.

J: If you do not help, he may die due to delayed treatment.

12. A person suffers a heart attack on a bus on your first day at work. After waiting a long time for the bus, he offers you money to hurry him to the hospital.

F: If you help, you risk losing a job that you cherish.

J: If you do not help, he may face life-threatening complications.

13. A person lies injured on the ground in front of an exhibition hall after being struck by a car, and the driver has fled. As a volunteer at the venue, he requests transportation to the nearest hospital.

F: If you help, you may not complete your assigned tasks on time, leading to significant issues for the exhibition.

J: If you do not help, he may face dangerous complications due to delayed treatment.

14. You witness a robbery occurring on the street as you pass by, and the victim calls out for help.

F: If you help, you may also become a victim of the robber and face retaliation.

J: If you do not help, he may lose his belongings and could be harmed.

15. A person has a flat tire on the highway, and a pregnant woman is in the car. You are on your way home to visit a sick relative when he waves for help.

F: If you help, it may take considerable time, and you may miss the opportunity to see your relative one last time.

J: If you do not help, the pregnant woman may not survive until assistance arrives.

16. A person bitten by a venomous snake is bleeding profusely. As a primary school teacher supervising students on a spring outing, he requests your help to reach the nearest hospital.

F: If you help, you cannot supervise the children, putting them at risk.

J: If you do not help, he may die due to delayed treatment.

17. A critically ill person is being rushed to the hospital by ambulance. As a firefighter responding to a fire alarm, the ambulance driver requests that you clear the way to expedite their arrival.

F: If you help, you may delay fire rescue efforts, worsening the situation.

J: If you do not help, he may die due to delayed treatment.

18. A person collapses from high blood pressure in a supermarket late at night. As a clerk working alone, he asks you to take him to the hospital for emergency care.

F: If you help, you risk losing your year-end bonus for leaving your post, and he will not compensate you.

J: If you do not help, he may die due to delayed treatment.

19. A person falls while crossing the street, losing his glasses and becoming immobilized. A truck is speeding toward him and cannot stop in time. He asks you to help find his glasses, but you are

the only one nearby.

F: If you help, both of you may be in danger.

J: If you do not help, he is likely to be struck by the truck.

20. A person calls for help from a second-floor window. As you pass by, you see a child falling from the balcony. He asks you to catch the child, and you are the only one who can reach him.

F: If you help, you may sustain injuries or even become disabled.

J: If you do not help, the child will fall to the concrete ground, with very low chances of survival.

21. A person falls into the water and, unable to swim, calls for help. You happen to be passing by, and he can only seek your assistance.

F: If you help, you may face life-threatening danger due to the cold water and your lack of experience.

J: If you do not help, he may not survive until help arrives.

22. A person with a mental illness seeks your assistance. As a renowned psychologist, you discover during the session that he has committed a serious crime. He asks you not to report him to the police.

F: If you help, his crime may go unpunished, potentially harming others.

J: If you do not help, you may lose your reputation for failing to maintain patient confidentiality and face retaliation.

23. During an important exam, you, as an invigilator, catch a student cheating. He pleads with you not to report him.

F: If you help by merely warning him, you may face disciplinary action for violating exam protocols.

J: If you do not help, he may lose an important opportunity and be banned from taking exams for three years.

24. A candidate accidentally leaves important materials in the waiting room during an interview. You, a fellow candidate, notice this, and he has a higher written test score than you. Will you help him return the materials?

F: If you help, your final score may be lower than his.

J: If you do not help, he may lose this job opportunity.

25. A person suffering from a rare disease comes from a poor family. As a pharmacist at a research institute, he requests that you sell him an expensive medication privately.

F: If you help, you may be fired for misusing public resources for personal gain.

J: If you do not help, he may continue to suffer from his illness.

26. A skier has an accident and is precariously hanging from an unstable cliff. He calls for help as you pass by, but rescuing him may put both of you at risk of falling.

F: If you help, you may face the danger of falling off the cliff.

J: If you do not help, he may not be able to hold on and could fall.

27. While climbing outdoors, a person's safety harness suddenly breaks, and he grabs your safety rope, which may not support both of your weights. He begs you for assistance.

F: If you help, both of you may fall.

J: If you do not help, you will have to cut the rope, resulting in his death.

28. An extreme sports enthusiast is skydiving with you when his parachute malfunctions and fails to open. He asks you to hold on tightly and not let go.

F: If you help, you may suffer severe hand injuries or disabilities due to the impact.

J: If you do not help, he may fall to his death due to excessive speed.

29. A person is robbed late at night by two men. You have just withdrawn a large sum of cash and are passing through an alley when he calls out for help.

F: If you help, you may also be robbed and lose your money.

J: If you do not help, he will certainly lose his money and may be injured.

30. A person slips and loses consciousness in the snow. You are on a business trip to the airport when he requests your assistance to reach the nearest hospital.

F: If you help, you may miss your flight and incur financial losses, and he will not compensate you.

J: If you do not help, he may freeze to death on the street due to prolonged unconsciousness.
